# Long term improvement in quality of life in Parkinson’s disease after deep brain stimulation of the subthalamic nucleus: an observational cohort

**DOI:** 10.1016/j.prdoa.2026.100481

**Published:** 2026-07-07

**Authors:** Ka Loong Kelvin Au, Vinay Pahwa, Kelly E. Lyons, Rajesh Pahwa

**Affiliations:** University of Kansas Medical Center, Department of Neurology, USA

**Keywords:** Parkinson's disease, Subthalamic nucleus, Deep brain stimulation, Long term outcomes, Quality of life

## Abstract

**Background:**

Long-term quality of life (QoL) outcomes following subthalamic nucleus (STN) deep brain stimulation (DBS) in Parkinson's disease (PD) remain incompletely characterized beyond 10–15 years.

Objective: To evaluate long term QoL and clinical outcomes in PD patients undergoing bilateral STN-DBS with follow up extending up to 25 years.

**Methods:**

Single center, prospective observational cohort study at a tertiary academic medical center. A total of 387 PD patients who underwent bilateral STN DBS were followed longitudinally with annual assessments for up to 25 years. Annual assessments included PDQ-39, Unified Parkinson's Disease Rating Scale (UPDRS), Levodopa equivalent daily dose (LED), Geriatric Depression Scale (GDS), Montreal Cognitive Assessment (MoCA) and Epworth Sleepiness Scale (ESS).

**Results:**

Among 387 patients (baseline PDQ-39, 35.34 ± 14.79), QoL improved significantly at year 1 (24.38 ± 14.39; *p* < 0.001) and year 3 (29.84 ± 15.70; p < 0.001), followed by stabilization at or near baseline through 25 years without sustained worsening. Motor function (UPDRS Part III, OFF state) improved significantly through year 12 (*p* < 0.001), with sustained reductions in OFF time and dyskinesia through year 20 (*p* < 0.001). LED was reduced by approximately 50% and remained significantly lower through year 19 (*p* < 0.001). Cognitive scores declined over time, with significant worsening beginning at year 2 (*p* < 0.03), and depression scores worsened gradually after year 3 (*p* = 0.04). Sleepiness improved transiently through year 7 (*p* = 0.02) before returning to baseline. PDQ-39 subscores showed domain-specific patterns, with sustained worsening observed in communication.

**Conclusion:**

Bilateral STN DBS is associated with significant early improvements in QoL followed by long-term stabilization without deterioration below baseline for up to 25 years. These findings suggest that STN DBS provides durable symptomatic benefit and relative preservation of QoL compared with the expected progressive decline in PD. Long-term management should prioritize non-motor symptoms, particularly cognition and communication.

## 1 Introduction

Quality of life (QoL) is an important health outcome measure for individuals undergoing subthalamic nucleus (STN) deep brain stimulation (DBS) for Parkinson's disease (PD). The Parkinson's Disease Questionnaire-39 (PDQ39) is a validated instrument for assessing patient reported health-related QoL in PD [1]. Prior studies have demonstrated sustained QoL improvements 5–15+ years following bilateral STN DBS [2], [3], [4]. Although QoL typically improves after DBS, progression of PD over time can lead to deterioration in QoL beyond 5–10 years in some cohorts [5], [6], [7], [8]. The natural history of PD is characterized by progressive decline in quality of life over time, whether STN-DBS alters this long-term trajectory beyond short-term improvements remains incompletely understood.

We previously reported outcomes at a mean follow up of 28 months and have since continued longitudinal follow-up of this cohort [9], [10]. To our knowledge, this study represents the the largest and longest follow-up of STN DBS outcomes, extending up to 25 years. These findings contribute to the limited data on long-term outcomes (1–3 decades) after STN DBS surgery and may inform counseling of prospective patients regarding expectated long-term outcomes.

## 2 Methods

We conducted a single center nonrandomized, open-label, prospective observational study of bilateral STN DBS in PD. All patients were deemed appropriate DBS candidates following standard neuropsychological and cognitive assessments. Levodopa responsiveness was confirmed using Unified Parkinson's Disease Rating Scale (UPDRS) Motor scores in the medication OFF and ON medication states. In a small subset, levodopa was not tolerated and only OFF-state scores were available.

All patients provided written informed consent, for use of their clinical data. The study was approved by the University of Kansas Medical Center Institutional Review Board-Human Subjects Committee (HSC 12351). There was no external funding for this study.

A total of 387 patients underwent bilateral STN DBS and were followed annually for up to 25 years. Outcome measures included PDQ-39 [11] and its subscores (Mobility, Activities of Daily Living (ADL), Emotional Well-being, Stigma, Social Support, Cognition, Communication, Bodily Discomfort), UPDRS [12] (OFF state), Montreal Cognitive Asessment (MoCA) [13], Geriatric Depression Scale (GDS) [14], Epworth Sleepiness Scale (ESS) [15] and Levodopa equivalent daily dose (LED) [16].

In individuals where MoCA testing was discontinued due to extremely low scores, prior scores were carried forward to subsequent visits to account for missing data (eg. A score of 15 at year 6 was applied to year 7 if reassessment could not be performed and if the prior MoCA was ≤10 we did not perform a repeat MoCA).

UPDRS Part II (ADL) scores were assessed in the medications OFF-state. Motor complications were evaluated by using UPDRS Part IV, including duration of OFF periods and dyskinesia. All comparisons were made relative to baseline values.

### 2.1 Statistical analysis

Data were analyzed using R version 4.4.1. Continuous variables are presented as means ± standard deviations (SD) and corresponding sample size (N). Longitudinal comparisons were performed using paired *t*-tests, with reported *p*-values.

Clinical outcomes at each annual follow-up visit were compared with pre-operative baseline values (year 0) using paired *t*-tests. To account for multiple comparisons across longitudinal follow-up time points, *p*-values were adjusted using the Benjamini–Hochberg false discovery rate (FDR) procedure. FDR correction was applied separately for each outcome measure. Significance was assessed using a threshold of α = 0.05 applied to the adjusted *p*-values.

Repeated-measures analyses were not performed because of the extended follow-up duration, irregular assessment intervals, and increasingly missing data over time resulting from loss to follow-up. The selected approach allowed direct assessment of change from baseline at each follow-up year while maintaining control of the false discovery rate. Graphical data include 95% confidence interval. Missing data were not imputed.

## 3 Results

**Fig. 1:**
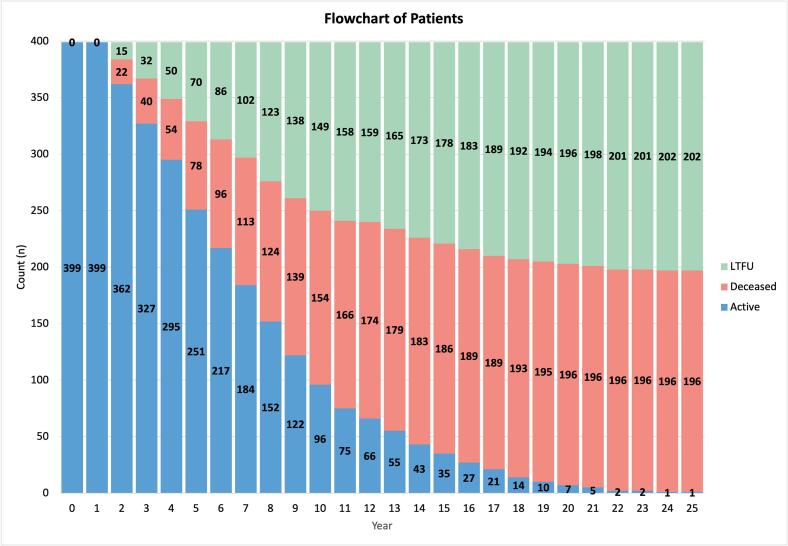
Flowchart of patients.

Baseline characteristics of our patient cohort are shown in Table 1. Over 25 years, attrition occurred due to loss to follow-up and mortality (Fig. 1).

**Table 1:** Baseline characteristics.

Clinical values	Mean ± SD (N)
Age at time of DBS surgery	62.94 ± 9.41 (399)
Age at PD onset	51.94 ± 9.90 (399)
Disease duration at time of DBS surgery (y)	11.03 ± 5.01 (399)
Sex (male / female)	66.9% / 33.1%
PDQ39 Total Score	35.34 ± 14.79 (382)
Mobility	48.06 ± 23.64 (382)
ADL	40.61 ± 21.16 (382)
Emotional Well-being	27.25 ± 17.99 (382)
Stigma	27.41 ± 22.09 (382)
Social Support	14.16 ± 17.57 (382)
Cognition	27.70 ± 19.21 (382)
Communication	29.49 ± 20.64 (382)
Bodily Discomfort	46.38 ± 22.52 (382)
UPDRS ADL OFF	20.10 ± 6.04 (387)
UPDRS ADL ON	10.29 ± 5.98 (381)
UPDRS Motor OFF	42.41 ± 9.16 (398)
UPDRS Motor ON	23.72 ± 8.08 (391)
OFF Duration	1.47 ± 0.74 (386)
Dyskinesia Duration	1.22 ± 0.81 (386)
LED	1366.34 ± 653.85 (399)
GDS	8.16 ± 5.47 (159)
ESS	9.93 ± 5.00 (327)
MoCA	25.49 ± 3.11 (321)

### 3.1 PDQ39 total score

**Fig. 2:**
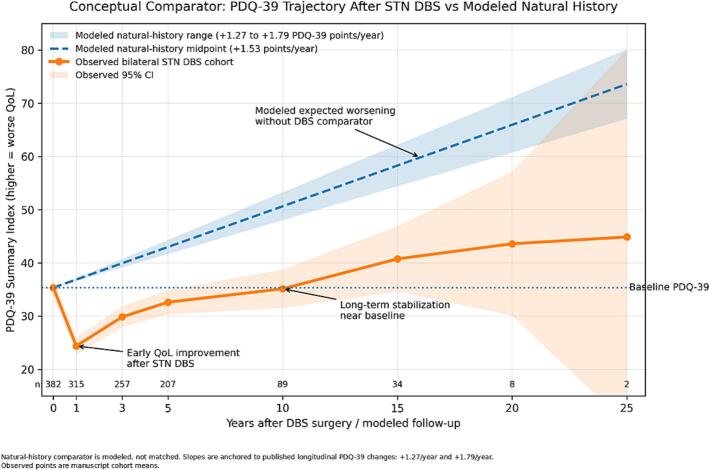
PDQ39.

**Fig. 3:**
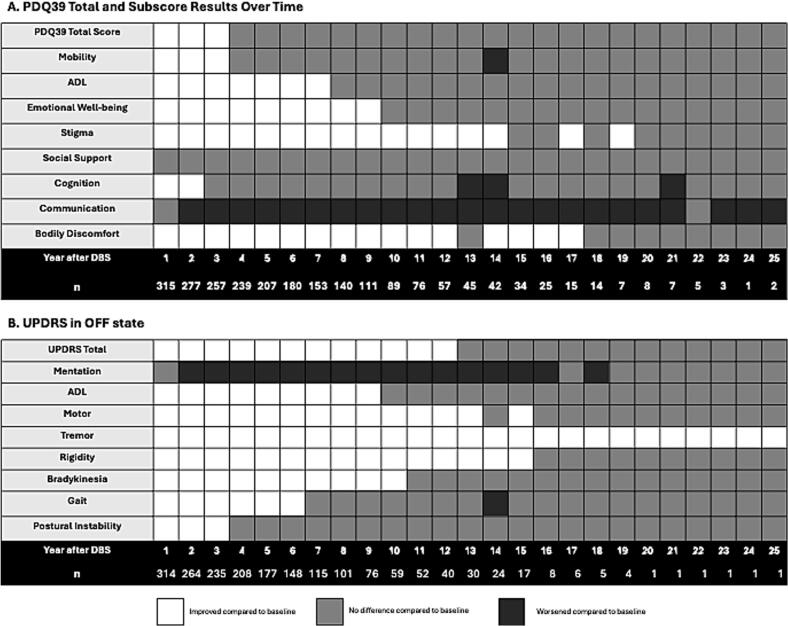
PDQ39 and UPDRS FDR corrected.

PDQ39 total scores improved significantly from baseline (35.34 ± 14.79; *n* = 382) at year 1 (24.38 ± 14.39, *p* < 0.001) and year 3 (29.84 ± 15.70, p < 0.001). From years 4–25, PDQ39 total scores were not significantly different compared from baseline (Fig. 2, Fig. 3A). We compared our observed PDQ39 total scores to a model comparator based on an average expected worsening of 1.53 (range 1.27–1.79) points per year (Fig. 2A) based on gradual expected worsening of quality of life in natural history studies [17], [18], [19], [20], [21], specifically with PDQ39 expected worsening by 1–2 points per year [18], [19], [22], [23].

### 3.2 PDQ39 subscores

Most PDQ-39 domains demonstrated early improvement followed by return to baseline (Fig. 3A):

**Mobility**: Improved through years 1–3, then returned to baseline by year 5.

**ADL:** Improved through years 1–5, then returned to baseline by year 10.

**Emotional Well-being and Stigma:** Sustained improvement essentially to year 10 before returning to baseline.

**Social Support:** No change compared to baseline throughout all years.

**Cognition:** Initial improvement for 2 years before returning to baseline, followed by decline beginning at years 13–14.

**Communication:** No improvement at year 1, progressive worsening began at year 2 which persisted.

**Bodily Discomfort:** Sustained improvement for approximately 15 years before returning to baseline.

### 3.3 UPDRS scores

**Total UPDRS (OFF state)** improved significantly through year 10 and returned to baseline by year 15 (Fig. 3B).

**Fig. 4:**
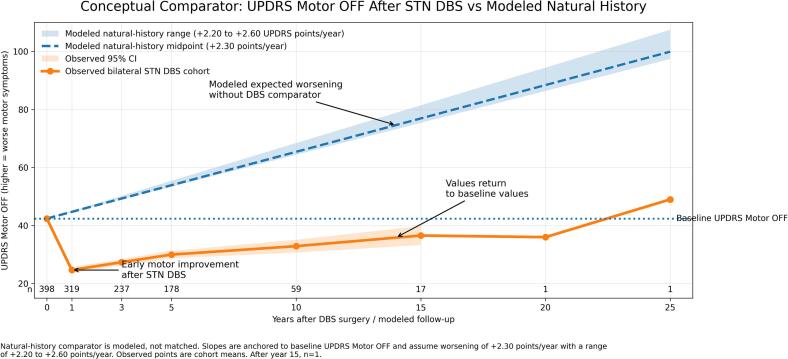
UPDRS OFF comparator final.

**UPDRS Part III OFF (Motor):** Sustained improvement in total motor score, bradykinesia and rigidity through year 15 followed by return to baseline. Tremor scores improved throughout all 25 years. Gait and postural stability only improved for 3–5 years before returning to baseline. Similar to the PDQ39 scores, the UPDRS Motor OFF scores were plotted against a model comparator using an expected average UPDRS Motor OFF worsening of 2.45 (range 2.30–2.60) annually (Fig. 4). Previous reports indicate UPDRS Motor OFF scores worsen 2.3–2.6 annually [24], [25].

**Fig. 5:**
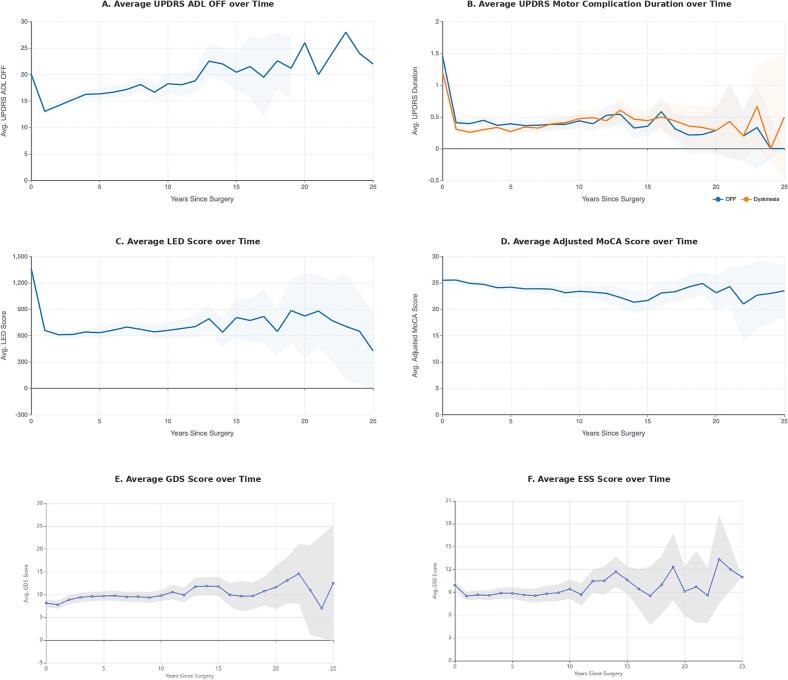
Combined scores.

**UPDRS Part II (ADL):** Improved through year 5, then returned to baseline by year 10 (Fig. 5A).

**UPDRS Mentation score:** No improvement at year 1 and progressive worsening began at year 2.

### 3.4 Motor complications

Both Off time and Dyskinesia duration were significantly reduced across all measured time points through year 20 (Fig. 5B).

### 3.5 Medications

LED was reduced by approximately 50% and remained significantly lower through year 20. After this, differences were no longer statistically significant, likely due to small sample size. (Fig. 5C).

Dopamine agonists, COMT inhibitors and anticholinergics showed the greatest reductions and were often discontinued. The use of antipsychotics, antidepressants and acetylcholinesterase inhibitors increased over time. (Supplemental Table 1).

### 3.6 Cognition

**Supplementary Fig. S1:**
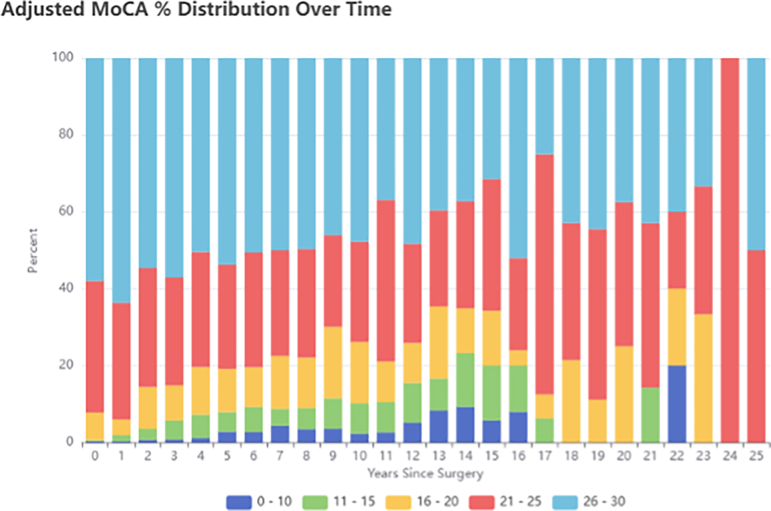
Adjusted MoCA distribution over time.

MoCA scores remained stable at year 1 but declined significantly from year 3 onward, consistent with disease progression (Fig. 5D). The percentage of individuals with a MoCA score greater than 26 steadily decreased over time (Supplemental Fig. 1).

### 3.7 Depression

GDS scores worsened gradually beginning at year 3 and remained elevated thereafter (Fig. 5E).

### 3.8 Sleepiness

ESS scores improved through year 5, then returned to baseline. (Fig. 5F).

## 4 Discussion

In this large, single-center longitudinal cohort, we report the longest follow-up to date of bilateral STN DBS in PD, with outcomes extending up to 25 years. We demonstrate that QoL, as measured by PDQ-39, improves during the first several years following DBS and subsequently stabilizes at or near baseline levels over the long term. Importantly, QoL did not show sustained deterioration below baseline despite the progressive nature of PD.

The natural history of PD is characterized by gradual worsening in QoL over time, driven by cumulative motor and non-motor symptom burden. Longitudinal studies suggest that PDQ-39 scores typically worsen by approximately 1–2 points per year, reflecting progressive disability [17], [18], [19], [20], [21], [23], [26], [27]. In contrast, our cohort demonstrated an initial improvement in QoL followed by long-term stabilization for up to 25 years after STN DBS. Although direct comparisons are limited by the absence of a non-DBS control group, this divergence from the expected trajectory suggests that STN DBS may provide sustained symptomatic benefit that attenuates, but does not halt, the impact of disease progression on QoL. These findings support the concept of relative preservation of QoL compared with the anticipated natural course of PD.

Our results are consistent with prior studies demonstrating early improvements in QoL after STN DBS, followed by variable long-term trajectories. Sustained improvement has been reported at 5–15 years in several cohorts [28], [29], [30], whereas other studies describe a return to baseline after 3–10 years [31], [32], [33]. Differences across studies likely reflect heterogeneity in patient populations, outcome measures, and duration of follow-up. Our cohort demonstrated short improvement over several years followed by return to baseline thereafter. By extending follow-up to 25 years, our study provides important insight into very long-term outcomes and suggests that stabilization, rather than progressive decline below baseline, may represent the dominant trajectory in patients who continue longitudinal follow-up.

Analysis of PDQ-39 subscores reveals important domain-specific patterns. Most domains, including mobility, activities of daily living, stigma, and emotional well-being, demonstrated early improvement followed by return to baseline. In contrast, communication showed progressive worsening beginning within a few years after surgery and continued to decline over time. This finding is consistent with prior reports of speech impairment associated with STN DBS and likely reflects a combination of disease progression and stimulation-related effects [34], [35]. Speech dysfunction remains a key unmet need in long-term DBS management and warrants targeted therapeutic strategies.

Cognitive outcomes followed a similar pattern, with stability in the first year followed by gradual decline over time. This trajectory parallels worsening MoCA scores and increased use of cognitive-enhancing medications, suggesting that cognitive decline is primarily driven by underlying disease progression rather than a direct effect of DBS [36]. However, our use of last observation carried forward for missing MoCA data may underestimate the degree of decline, and this should be interpreted with caution.

From a motor perspective, STN DBS provided robust and sustained benefit, with significant improvements in UPDRS motor scores in the medication OFF state lasting up to 15 years. Additionally, reductions in motor complications, including OFF time and dyskinesia, were maintained for up to 20 years. These findings are consistent with the established efficacy of STN DBS in improving motor symptoms and reducing motor fluctuations [2], [4], [28], [29]. The sustained reduction in levodopa equivalent daily dose (LED), particularly in the first 15 years, further supports the long-term therapeutic impact of DBS and may contribute to improved QoL by reducing medication-related adverse effects [37].

Non-motor outcomes demonstrated a more complex trajectory. Depression scores gradually worsened after the initial postoperative period, and sleepiness improved transiently before returning to baseline. These findings highlight the increasing contribution of non-motor symptoms to overall disease burden over time and underscore the importance of comprehensive long-term management beyond motor symptom control.

### 4.1 Study limitations

Several limitations should be considered. First, the absence of a non-DBS control group limits direct comparison with the natural history of PD. However, we address this through a conceptual comparator based on published longitudinal data. Second, attrition over time, due to loss to follow-up and mortality, likely introduces bias toward patients with better long-term outcomes, potentially overestimating sustained QoL benefits. Third, not all patients were able to undergo standardized ON and OFF medication assessments at each time point, and ON-state data were limited. Fourth, cognitive data were incomplete in later years, necessitating imputation strategies that may underestimate decline. Finally, imaging data regarding lead placement were not available, precluding analysis of the relationship between electrode location and clinical outcomes.

Despite these limitations, this study provides unique long-term data on the durability of STN DBS effects. Our findings suggest that while PD progression ultimately attenuates early gains in QoL, STN DBS may confer sustained benefit by stabilizing QoL relative to the expected trajectory of decline. This distinction is clinically meaningful and supports the role of DBS as a long-term symptomatic therapy.

## 5 Conclusion

Bilateral STN DBS results in significant early improvements in QoL, followed by long-term stabilization without deterioration below baseline for up to 25 years. These findings provide important context for counseling patients regarding long-term expectations and highlight the need for continued focus on non-motor symptoms, particularly cognition and communication, in the chronic management of PD following DBS.

The following are the supplementary data related to this article.

Supplementary Table S1 Medication class usage.

## Credit authorship contribution statement

**Ka Loong Kelvin Au:** Writing – review & editing, Writing – original draft, Data curation, Conceptualization. **Vinay Pahwa:** Visualization, Validation, Formal analysis, Data curation. **Kelly E. Lyons:** Writing – review & editing, Validation, Supervision, Project administration, Methodology, Investigation, Formal analysis, Data curation, Conceptualization. **Rajesh Pahwa:** Writing – review & editing, Validation, Supervision, Resources, Project administration, Methodology, Investigation, Formal analysis, Data curation, Conceptualization.

## Declaration of competing interest

The authors declare that they have no known competing financial interests or personal relationships that could have appeared to influence the work reported in this paper.
